# Assessing the quality of antimicrobial prescribing in solid organ transplant recipients: a new frontier in antimicrobial stewardship

**DOI:** 10.1017/ash.2024.49

**Published:** 2024-05-03

**Authors:** Sagar Kothari, Syed Z. Ahmad, Michelle T. Zhao, Abbigayle Teixeira-Barreira, Miranda So, Shahid Husain

**Affiliations:** 1 Transplant Infectious Diseases, Ajmera Transplant Center, University Health Network, Toronto, ON, Canada; 2 Temerty Faculty of Medicine, University of Toronto, Toronto, ON, Canada; 3 Sinai Health System-University Health Network Antimicrobial Stewardship Program, University Health Network, Toronto, ON, Canada; 4 Leslie Dan Faculty of Pharmacy, University of Toronto, Toronto, ON, Canada; 5 Division of Infectious Diseases, Faculty of Medicine, Dentistry and Health Sciences, University of Melbourne, VIC, Australia

## Abstract

**Background:**

Post-transplant infections remain a leading cause of morbidity and mortality in solid organ transplant recipients (SOTRs) and local standardized antimicrobial treatment guidelines may contribute to improved clinical outcomes. Our study assessed the rate of therapeutic compliance with local standard guidelines in the treatment of common infections in SOTR, and their associated outcomes.

**Methods:**

Consecutive adult SOTRs admitted to the transplant floor from January–September 2020 and were treated for an infectious syndrome were followed until discharge or for 30 days following the date of diagnosis, whichever was shorter. Data was extracted from electronic medical records. Guideline compliance was characterized as either appropriate, effective but unnecessary, undertreatment, or inappropriate.

**Results:**

Nine hundred and thirty-six SOTR were admitted to the transplant ward, of which 328 patients (35%) received treatment for infectious syndromes. Guidelines were applicable to 252 patients, constituting 275 syndromes: 86 pneumonias; 82 urinary tract infections; 40 intra-abdominal infections; 38 bloodstream infections; and 29 *C. difficile* infections. 200/246 (81%) of infectious syndromes received appropriate or effective but unnecessary empiric treatment. In addition, appropriate tailoring of antimicrobials resulted in a significant difference in 30-day all-cause mortality (adjusted OR of 0.07, 95% CI 0.01–0.38; *P* = .002). Lastly, we found that guideline-compliant empiric therapy was found to prevent the development of multi-drug resistance in a time-dependent analysis (adjusted HR of 0.21, 95% CI 0.08–0.52; *P* = .001).

**Conclusion:**

Our data show that adherence to locally developed guidelines was associated with reduced mortality and resistant-organism development in our cohort of SOTR.

## Introduction

Despite medical advancements leading to improved outcomes for solid organ transplant recipients (SOTR), post-transplant infections remain a leading cause of morbidity and mortality within this population^1^. Common syndromes encountered after transplantation include bloodstream, respiratory tract, genitourinary, hepatobiliary, and gastrointestinal infections, the diagnostic criteria of which are relatively well-defined^2^. The development of these infectious diseases is associated with adverse patient outcomes, such as increased rates of graft dysfunction and increased treatment costs, as well as the harms associated with antibiotic overuse^3,4^. Avoiding antimicrobial overuse in SOTR is necessary for preventing the development of multi-drug resistance/toxicity, including *Clostridium difficile* infection, which can subsequently lead to graft loss and an increased risk of mortality post-transplant^5,6^. These outcomes underscore the importance of applying antimicrobial stewardship guidelines for the optimal management of post-transplant infectious syndromes.

Established guidelines suggest that antimicrobial therapy should initially be empiric, relying on clinical presentation and employing specific recommended broad-spectrum agents to treat multiple suspected pathogens before treatment is de-escalated once microbiological data is made available. Despite the existence of these guidelines, the antimicrobial treatment of each syndrome is subject to wide variation. Studies examining rates of guidelines-compliant therapy have found adherence to be in the range of 43.5%–59%^7–9^. Prescribers not implementing guideline-compliant treatment have been found to prescribe broad-spectrum therapy more often, which may not always be necessary, contributing to already increasing rates of multi-drug bacterial resistance^10,11^.

While there are many available stewardship guidelines, though variable across patient populations and location, few exist for the SOT population specifically. Furthermore, it is unclear if these guidelines are effective in this patient population. Controversy exists as to whether complying with guidelines is associated with improved patient outcomes^12–15^. Some studies have shown that adherence to guidelines does not have a significant impact on patient outcomes such as mortality and length of hospital stay^12,16^. Conversely, other papers explicitly oppose these findings by concluding that guideline compliance is associated with improved patient outcomes such as an increased quality of life and a decreased patient length of stay, as well as decreased treatment costs^9,10,12,13,17–24^. Despite these differing conclusions, most of the research dissenting on adherence to specific guidelines agree that ultimately a narrower spectrum of antimicrobial therapy should be utilized^12^.

Due to the distinct lack of consensus within the scientific community regarding the clinical significance of guideline adherence, as well as the need for studies centered on the SOT population, this study will provide much-needed validation of the efficacy of current treatment guidelines within this patient group in Canada.

## Methods

### Study design and outcomes

This retrospective cohort study enrolled consecutive adult SOTR admitted to the transplant floor who received treatment for an infectious syndrome at the Toronto General Hospital, Toronto, Ontario, Canada, from January to September 2020. Patients must have had an infectious syndrome to which management guidelines were applicable (bloodstream, respiratory tract, intra-abdominal, urinary tract infections, or first episodes of *C. difficile* infections) to be included. Patient admitted for transplantation who did not develop a qualifying infection post-transplant were excluded. We also excluded those whose treatment was initiated in an intensive care unit (ICU) or at another institution. A patient could only be included once during the study period but could be included for multiple infections within the admission. Patients were followed until discharge or for 30 days following the date of diagnosis, whichever was shorter. The study was approved by the institutional research ethics board (REB: 20-5029).

Clinical data was extracted from electronic medical records and included information on demographics, transplant type, antimicrobial use, consultations, laboratory and radiological investigations, as well as length of stay and ICU admission. The primary outcome was the rate of compliance with local standard guidelines for empiric and tailored management of the infectious syndrome. Secondary outcomes included rejection, *C. difficile* infection, graft loss, re-admission, and death within 30 days. We also created a composite outcome of either (1) mortality, (2) ICU admission, (3) graft rejection or loss, and (4) readmission, wherein patients with more than one infectious syndrome were only considered once.

### Transplant program

The Ajmera Transplant Center at the Toronto General Hospital performs more than 600 solid organ transplants every year. The study period was expected to capture 200–250 qualifying infectious episodes given the incidence of infection is estimated to be 40%. This study was initiated in January 2020, and due to effects of the COVID-19 pandemic, was completed in September 2020.

### Local standard guidelines

Development of the guidelines was done by the Antimicrobial Stewardship Program of the institution and was specific for solid organ transplant recipients^25^. These guidelines were based on local microbiological data from SOTR, current literature, and other published guidelines. The guidelines recommend empiric carbapenems and daptomycin due to local prevalence of multi-drug-resistant gram-negative rods and vancomycin-resistant enterococci, with strong emphasis on tailoring therapy. The guidelines can be assessed at (https://www.antimicrobialstewardship.com/infectioninsot)

### Definitions of compliance

We categorized guideline compliance as either inappropriate, under-treatment, appropriate, or effective but unnecessary, based on definitions by Dresser and colleagues^26^. Briefly, criteria for inappropriate included the use of antimicrobials for pre-emptive therapy without evidence to support the practice, for under-treatment included prescription of antimicrobials with insufficient activity to treat the causative organism, and for effective but unnecessary included therapy that has too broad a spectrum of activity.

### Statistical analysis

Categorical variables were compared using the χ2 test and Fisher’s exact test as appropriate. The Mann-Whitney *U* test was used to determine the association between continuous variables and study outcomes. We considered *P* < .05 as the level of statistical significance. To assess the association between appropriate empirical antimicrobial treatment and study outcomes, we conducted univariable and multivariable logistic regression for binary outcomes and linear regression for length of stay. Cox regression was used to model the association between receipt of guideline-compliant (appropriate or effective but unnecessary) therapy and the time to emergence of drug-resistant bacterial infections in the study period, as compared to therapy that was not guideline-compliant (undertreatment or inappropriate). We adjusted for age (<65 vs ≥65 yr), sex, and Charlson comorbidity index. All analyses were conducted using STATA® version 16.1 (College Station, TX, USA).

## Results

**Figure 1.**
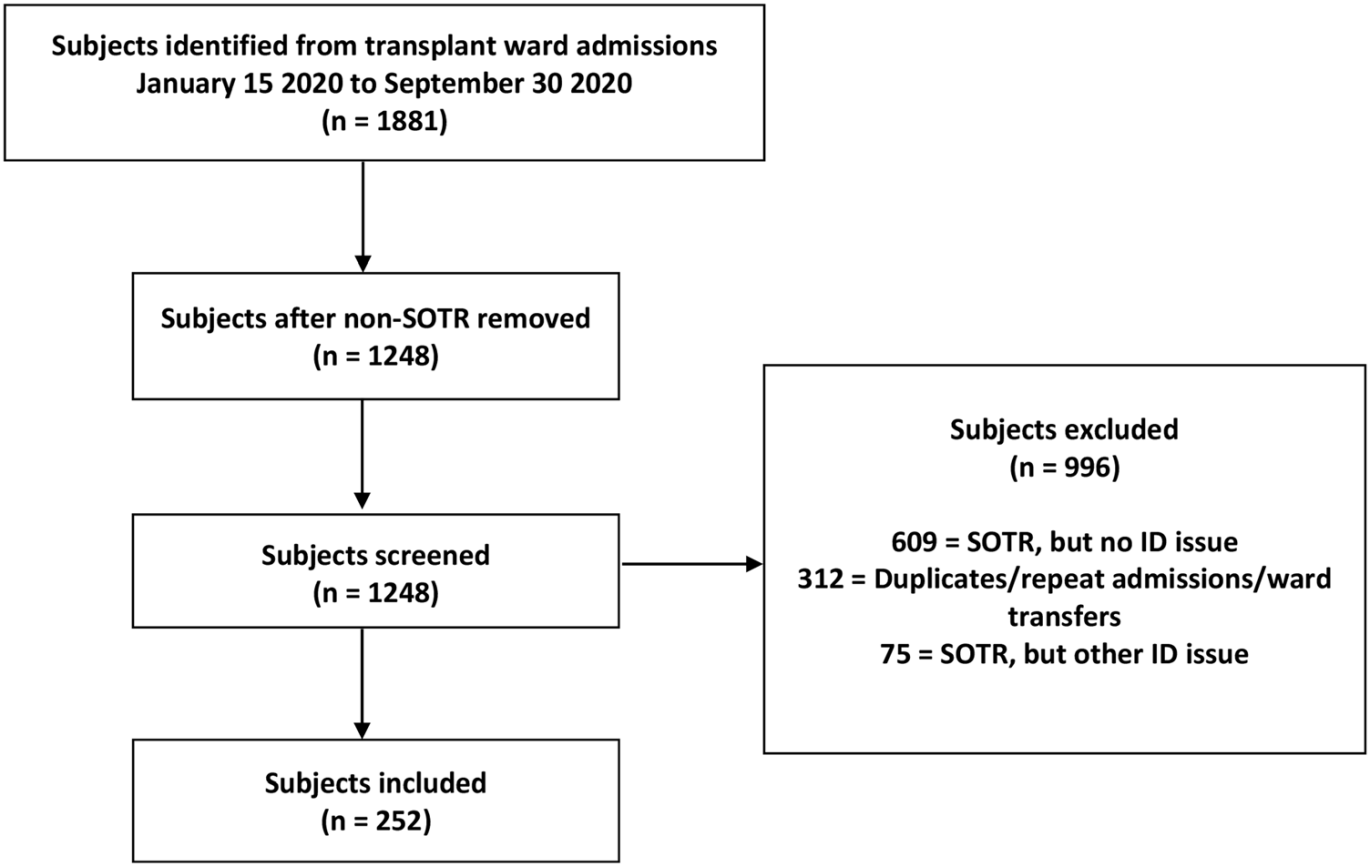
Study Flow Diagram.

Nine hundred and thirty-six SOTR were admitted to the transplant ward, of which 328 patients (35%) were admitted with infectious syndromes. Guidelines were applicable to 252 patients, constituting the following 275 syndromes: 86 pneumonias; 82 urinary tract infections (UTI); 40 intra-abdominal infections (IAI); 38 bloodstream infections; and 29 C. difficile infections (Figure 1 and Table 1).

**Table 1.** Cohort Demographics at Baseline

Organ	Number of patients (n, %)	Male (n, %)	Median age, IQR (years)	Mean time since transplant (months)	Induction ATG (n)	Induction basiliximab (n)	Mean CCI	Pneumonia (n, %)	Bacteremia (n, %)	UTI (n, %)	Intra-abdominal (n, %)	C-difficile infection (n, %)
Total	252 (100)	143 (56.8)	60 (20)	75	7	33	2.6	86 (31.3)	38 (13.8)	82 (29.8)	40 (14.5)	29 (10.5)
Kidney	85 (33.6)	43 (50.6)	60 (18)	105	4	12	2.4	17 (18.5)	10 (10.9)	48 (52.2)	8 (8.7)	9 (9.8)
Liver	68 (27.0)	42 (61.8)	57 (18)	52	0	14	2.6	13 (18.1)	16 (22.2)	13 (18.1)	20 (27.8)	10 (13.9)
Lung	66 (26.2)	39 (59.1)	64 (23)	47	2	4	3.0	46 (63)	6 (8.2)	13 (17.8)	3 (4.1)	5 (6.8)
Kidney-Pancreas	16 (6.4)	9 (56.3)	53 (16)	112	1	3	1.6	3 (18.8)	2 (12.5)	4 (25)	6 (37.5)	1 (6.3)
Heart	12 (4.8)	8 (66.7)	60.5 (12)	69	0	0	2.7	6 (37.5)	2 (12.5)	3 (18.8)	2 (12.5)	3 (18.8)
Other	4 (1.6)	1 (25)	51.5 (20)	145	0	0	2.5	1 (20)	1 (20)	1 (20)	1 (20)	1 (20)
Pancreas after Kidney	1 (0.4)	1 (100)	48 (0)	111	0	0	2	0 (0)	1 (100)	0 (0)	0 (0)	0 (0)

Note. Other = other combination of multiorgan transplantation (heart/kidney, liver/pancreas, kidney/liver, lung followed by kidney/pancreas); IQR = Interquartile range.

Baseline demographics of the cohort are described in Table 1. Fifty-seven percent of study participants were male and most patients were beyond 1-year post-transplant, with a mean time of 75 months post-transplant. Most patients had an infectious syndrome in their transplanted organ. Sixty-three percent of pneumonia occurred in lung transplant recipients, 78% of UTIs occurred in patients with a kidney allograft, and 74% of IAI occurred in abdominal organ transplant (kidney, kidney-pancreas, or liver) recipients. The mean Charlson Comorbidity Index across the cohort was 2.6.

### Compliance with guidelines

Guideline-compliant empiric prescribing varied by infectious syndrome (Table 2). In total, 92% of pneumonia were treated in concordance with guidelines (85% appropriate and 7% effective but unnecessary). Under-treatment was rare (8%), usually with a third-generation cephalosporin instead of piperacillin-tazobactam. Of the 7% of cases that received effective but unnecessary treatment, most received atypical organisms’ coverage in addition to routine recommendations.

**Table 2.** Prevalence of guideline-compliant empirical prescribing by clinical syndrome

Infectious syndrome	Guideline compliant		Guideline non-compliant			Total
	Appropriate	Effective-unnecessary	Under-treatment	Inappropriate	Not assessable	
Pneumonia	73 (84.9%)	6 (7.0%)	7 (8.1%)	0	0	86
Intra-abdominal infection	20 (50%)	7 (17.5%)	11 (27.5%)	1 (2.5%)	1 (2.5%)	40
Urinary tract infection	45 (54.9%)	29 (35.4%)	8 (9.8%)	0	0	82
Bacteremia	13 (34.2%)	7 (18.4%)	15 (39.5%)	0	3 (7.9%)	38

For intra-abdominal infections, 50% were treated appropriately, 17.5% effective but unnecessary, and 27.5% were under-treated. Under-treatment was usually seen through the lack of empiric agents active against previously isolated multi-drug-resistant organisms; in one case, no antibiotics were administered. Effective but unnecessary treatment was commonly the addition of vancomycin to piperacillin-tazobactam or the usage of carbapenems in the absence of a history of multi-drug-resistant organisms.

For UTIs, 55% of treatment regimens were classified as appropriate, 35% as effective but unnecessary, and 10% as undertreatment. Effective but unnecessary treatment was commonly the use of piperacillin-tazobactam or a carbapenem, instead of ceftriaxone. Undertreatment was most frequently attributed to the absence of antimicrobials in symptomatic bacteriuria.

For bacteremia, 34% were treated appropriately, 18% were effective but unnecessary, and 40% were undertreated. Use of piperacillin-tazobactam was the most common reason for undertreatment (instead of meropenem) and for effective but unnecessary treatment as well (instead of vancomycin).

Empiric prescribing for *C. difficile* infection was unable to be assessed as no patients received empiric therapy; however, all patients with this syndrome received guideline-compliant tailored therapy. Reasons for effective but unnecessary treatment were commonly the improper continuation of proton pump inhibitors, H2-receptor antagonists, or anti-peristaltic agents.

### Outcomes and compliance

Regarding empirical therapy, overall, we did not identify any difference in outcomes between patients who received guideline-compliant therapy with those who did not. The lack of association was observed in both the unadjusted and adjusted logistic regression models (Table 4 and Supplementary Appendix). Similar patterns were seen at the syndrome level. For tailored therapy, overall, we observed that receipt of guideline-compliant therapy was protective against all-cause mortality at 30 days (adjusted OR of 0.07, 95% CI 0.01–0.38; *P* = .002). This finding was consistent in both the unadjusted and adjusted analyses (Table 5 and Supplementary Appendix). However, we did not identify any significant between-group differences in the other clinical outcomes. Similar patterns were seen at the syndrome level.

**Table 3.** Prevalence of outcome events by empirical guideline concordance category

Outcome	Guideline compliant		Guideline non-compliant		Not assessable (n = 4)	Total (n = 234)
	Appropriate (n = 143)	Effective- unnecessary (n = 46)	Under-treatment (n = 40)	Inappropriate (n = 1)		
ICU admission (n = 33)	14.0% (20/143)	10.9% (5/46)	20.0% (8/40)	0	0	14.1% (33/234)
Graft rejection within 30 days (n = 3)	1.4% (2/143)	0	2.5% (1/40)	0	0	1.3% (3/234)
Graft loss within 30 days (n = 4)	2.1% (3/143)	0	2.5% (1/40)	0	0	1.7% (4/234)
CDI within 30 days (n = 1)	0.7% (1/143)	0	0	0	0	0.4% (1/234)
Readmission within 30 days (n = 77)	35.0% (50/143)	30.4% (14/46)	30% (12/40)	0	25% (1/4)	32.9% (77/234)
Mortality at 30 days (n = 11)	4.2% (6/143)	2.2% (1/46)	10% (4/40)	0	0	4.7% (11/234)

**Table 4.** Multivariable Regression of Outcomes, by Compliance with Empiric Therapy (Compliant vs reference)

	All Syndromes (n = 230)	Pneumonia (n = 86)	Intra-abdominal (n = 39)	UTI (n = 82)	Bacteremia (n = 35)
	aOR (95% CI)	aOR (95% CI)	aOR (95% CI)	aOR (95% CI)	aOR (95% CI)
ICU admission	0.64 (0.27–1.55), *P* = .322	1.19 (0.13–11.28), *P* = .877	0.16 (0.01–2.28), *P* = .178	0.24 (0.04–1.69), *P* = .153	0.97 (0.17–5.58), *P* = .974
Graft rejection within 30 days	0.41 (0.03–4.87), *P* = .482	–	–	–	0.76 (0.04–15.79), *P* = .858
Graft loss within 30 days	0.68 (0.07–7.05), *P* = .745	–	–	–	–
CDI with 30 days	–	–	–	–	–
Re-admission within 30 days	1.15 (0.56–2.37), *P* = .708	0.50 (0.10–2.53), *P* = .402	9.60 (0.99–92.83), *P* = .051	0.64 (0.14–2.90), *P* = .566	3.28 (0.72–15.03), *P* = .125
Mortality at 30 days	0.33 (0.09–1.23), *P* = .099	0.17 (0.02–1.37), *P* = .096	–	–	0.77 (0.07–9.39), *P* = .857
Composite outcome^a^	0.74 (0.38–1.50), *P* = .388	0.41 (0.08–2.09), *P* = .286	1.50 (0.33–6.86), *P* = .602	0.22 (0.04–1.22), *P* = .083	3.81 (0.80–18.19), *P* = .094
Hospital LOS	8.23 (–13.38–29.84), *P* = .454	–11.03 (–45.7–23.60), *P* = .528	13.56 (–17.83–44.95), *P* = .386	9.54 (–54.76–73.84), *P* = .769	48.2 (–4.09–100.4), *P* = .069

a Patients with more than one infectious syndrome were counted once (n = 234), but model was n = 230 as 4 were non-assessable.

**Table 5.** Multivariable Regression of Outcomes, by Compliance with Tailored Therapy (Compliant vs reference)

	All syndromes combined (n = 252)	Pneumonia (n = 86)	Intra-abdominal infection (n = 40)	UTI (n = 82)	Bacteremia (n = 38)	*C. difficile* infection (n = 29)
	aOR (95% CI)	aOR (95% CI)	aOR (95% CI)	aOR (95% CI)^a^	aOR (95% CI)^a^	aOR (95% CI)
ICU admission	0.51 (0.13–1.97), *P* = .328	0.67 (0.06–8.09), *P* = .754	–	1.02 (0.10–10.36), *P* = .990	0.15 (0.01–3.07), *P* = .216	–
Graft rejection within 30 days	–	–	–	–	–	–
Graft loss within 30 days	–	–	–	–	–	–
CDI with 30 days	–	–	–	–	–	–
Re-admission within 30 days	1.25 (0.38–4.18), *P* = .708	1.24 (0.11–13.94), *P* = .864	–	1.02 (0.10–10.36), *P* = .990	1.72 (0.12–23.70), *P* = .685	–
Mortality at 30 days	**0.07 (0.01–0.38),** ***P*** **= .** **002**	0.14 (0.01–2.56), *P* = .186	–	–	–	–
Composite outcome	0.70 (0.24–2.09), *P* = .526	0.45 (0.05–3.74), *P* = .458	–	2.12 (0.37–11.99), *P* = .396	–	–
Hospital LOS	–0.4 (–34.1–33.3), *P* = .981	14.96 (–31.10–61.02), *P* = .520	–	–6.88 (–74.94–61.18), *P* = .841	46.54 (–48.54–141.62), *P* = .327	–

a Adjusted for age and sex only. Odds ratios were unable to be created when the sample size between the two categories (compliant vs non-compliant) was too low. Bold values denote statistical significance at the *p* < .05 level.

**Figure 2.**
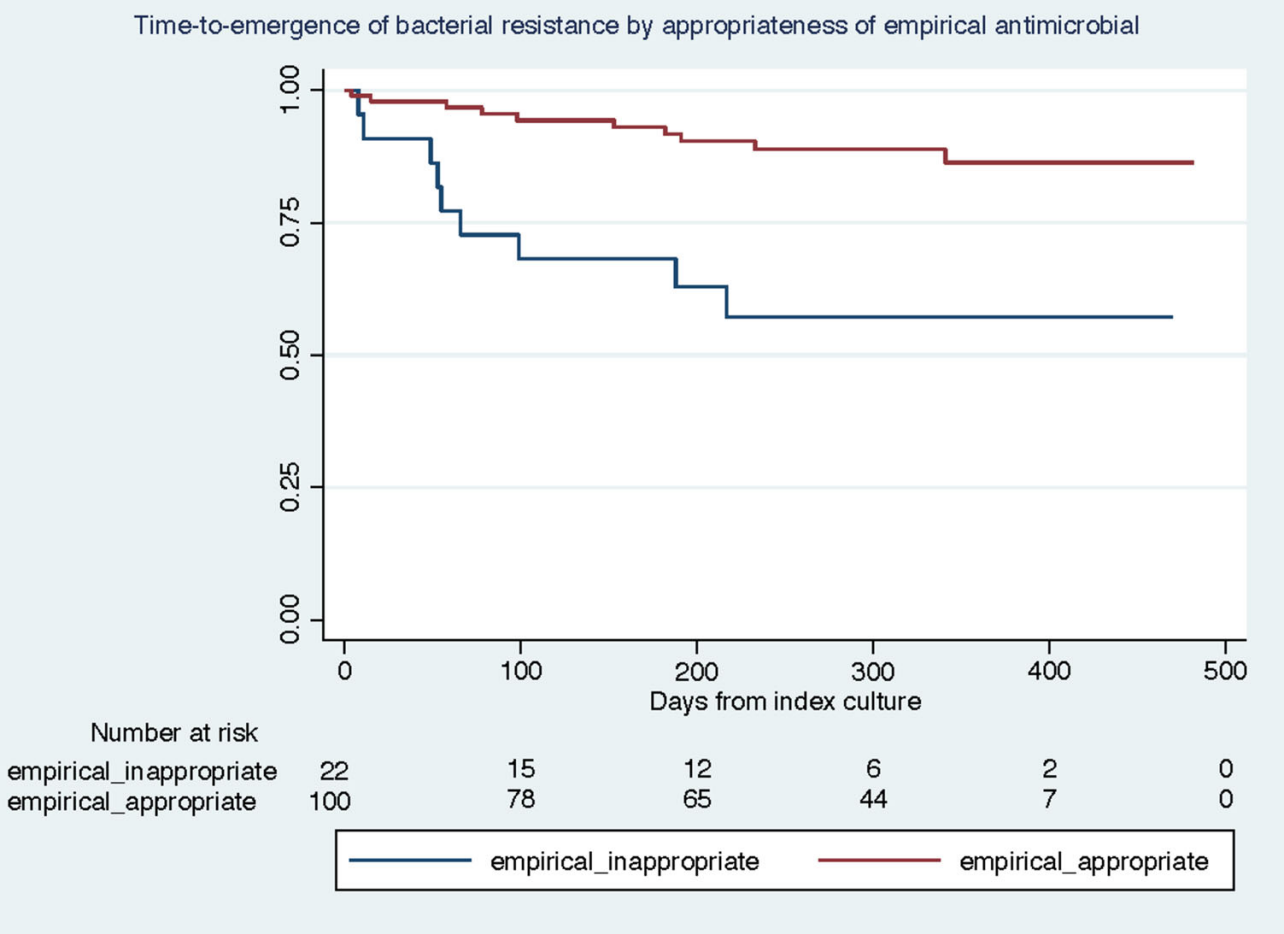
Kaplan-Meier estimates of guideline compliant versus non-compliant empiric therapy on development of resistance.

Among patients who had a positive bacterial culture at the index microbiology result, guideline-compliant empirical therapy was associated with lower odds of emergence of an MDR bacterial isolate in both unadjusted and adjusted analyses. The adjusted odds ratio, comparing guideline compliant with non-compliant empirical antibiotics, was 0.14 (95% CI 0.04–0.43; *P* = .001). Moreover, in a time-to-event analysis, guideline-compliant empiric therapy was associated with a lower risk of developing an MDR isolate within one year (adjusted HR of 0.21, 95% CI 0.08–0.52; *P* = .001) (Figure 2).

## Discussion

The development of guidelines to standardize management of common infectious syndromes which are tailored to local epidemiology and facility-specific characteristics is an important component of the antimicrobial stewardship toolkit^27–29^. Under the auspices of quality improvement, evaluating adherence to such guidelines can confirm their validity, in addition to informing knowledge translation and implementation strategies. SOTR are disproportionately burdened by antimicrobial resistance, antimicrobial-related adverse events, and healthcare-associated infections^30,31^. Local guidelines addressing their unique antimicrobial needs may facilitate appropriate prescribing practices. Our study assessed guideline adherence and further evaluated clinical outcomes according to appropriateness of antimicrobial use. We found a high overall adherence to guidelines at our center as compared to other studies, and that appropriate antimicrobial tailoring was associated with lower mortality. In addition, we found that guideline-compliant empiric therapy was found to prevent the development of multi-drug resistance in a time-dependent analysis.

In our cohort, guideline-compliant antimicrobial prescribing was high. 200/246 (81%) of infectious syndromes received appropriate or effective but unnecessary empiric treatment (Table 2). While difficult to compare our data to non-SOT studies, most reports such as those from Hagen et al., have found adherence to clinical guidelines to be lower^8^. In Hagen’s work, only 53% of patients with community-acquired infections received guideline-compliant antimicrobial therapy. We believe that the higher compliance with compliant therapy was the result of our biweekly audit and feedback ASP program, as described in a previous paper detailing the implementation of an antimicrobial stewardship intervention^30^.

We also observed that patients who received guideline-compliant empiric therapy had a lower risk (aHR 0.21, *P* = .001) of developing an MDR isolate within one year. While there are many studies demonstrating that antimicrobial stewardship programs are an effective tool at combating bacterial resistance, this is the first report to our knowledge in SOT that provides evidence of effectiveness in the SOT population specifically^32–35^. Local stewardship programs aimed at optimizing the prescription of antimicrobials can guide prescribing practices while retaining the autonomy of the prescribers and may help prevent adverse outcomes such as the development of difficult-to-treat pathogens.

While we found that bloodstream infections had a high rate of non-compliant empiric therapy (48%), this did not result in any observable differences in outcomes. Though empiric choice of antimicrobial is undoubtedly important to ensure adequate coverage, most of our undertreated patients received piperacillin-tazobactam instead of meropenem. Owing to the lower prevalence of MDR gram-negative organisms seen, they might have received adequate therapy, which may have contributed to the lack of difference in mortality. However, we found that appropriate tailoring of antimicrobials resulted in a large difference in 30-day all-cause mortality from diagnosis (aOR of 0.07, *P* = .002). To our knowledge, this is the first study in the SOT population to report that tailoring of antimicrobials could be associated with improved survival. In a non-SOT context, Crowell et al. found improved that treatment compliance with standardized (Infectious Diseases Society of America/The Society for Healthcare Epidemiology of America) guidelines for the treatment of *C. difficile* infection was associated with a decreased risk of mortality and LOS^36^. Our findings are consistent with other studies emphasizing the importance of tailoring antimicrobials appropriately, which prevents the adverse events associated with antibiotic overuse^37–39^.

There are limitations to this study. This was a single-center study conducted at a large transplant center, which only included retrospective data from inpatients. The lack of clear documentation for reasoning of the choice of antibiotic may contribute to confounding. We made our best effort to mitigate against potential biases by using a pre-specified framework, as well as microbiological data, radiological reports, and drug administration records to confirm treatment rationale. Our study was not designed to challenge the diagnosis made by treating physicians; hence, we were unable to ascertain the appropriateness of antibiotic therapy for asymptomatic bacteriuria. However, we have well established and disseminated guidelines stressing not to treat patients with asymptomatic bacteriuria. Additionally, despite a reasonably sized cohort, (n = 252), outcomes occurred at a low frequency. It is likely that our study was underpowered to detect differences between groups due to the low incidence of adverse events. Future studies with larger patient cohorts across multiple centers may be helpful. Finally, while compliance was assessed according to the ASP guidelines and published definitions^26^, we acknowledge that these standardized definitions were created using the Delphi method for the critical care setting, not specifically for SOTR. Nonetheless, this study was a launching point for assessing the quality of antimicrobial use in SOTR^40^.

Overall, our data show that guidelines for the management of infectious syndromes are of paramount importance, especially in the SOT population. Adherence to guidelines resulted in reduced mortality and resistant-organism development in our cohort, though this finding should be validated in larger multi-center studies. Efforts that focus on adherence to these guidelines may improve outcomes in this patient population.

## Supporting information

Kothari et al. supplementary material Kothari et al. supplementary material

## Data Availability

The data presented in this study are available upon reasonable request from the corresponding author.

## Supplementary material

For supplementary material accompanying this paper visit http://doi.org/10.1017/ash.2024.49. click here to view supplementary material
