# Supplementary material for: Assessing the quality of antimicrobial prescribing in solid organ transplant recipients: a new frontier in antimicrobial stewardship
Source: Antimicrob Steward Healthc Epidemiol. 2024 May 3;4(1):e72. doi: 10.1017/ash.2024.49 (PMC11094401; doi:10.1017/ash.2024.49)
Supplement: Kothari et al. supplementary material [file S2732494X24000494sup001.docx]

Supplementary Appendix

**Title:** Assessing the Quality of Antimicrobial Prescribing in Solid Organ Transplant Recipients: A New Frontier in Antimicrobial Stewardship

**Authors:** Sagar Kothari, HBSc^1,2*^, Syed Z. Ahmad, HBSc^1,2*^, Michelle T. Zhao, HBSc^1,2^, Abbigayle Teixeria-Barreira, HBSc^1^, Miranda So, PharmD MPH^3-5^, Shahid Husain, MD^1-3^

Tables 4 and 5 in the main text include solely the adjusted analyses, however we do wish to provide the results of the unadjusted analyses in tandem, for benefit of the readers. Please find the results below.

**Outcomes, empirical therapy:**

**All syndromes, empirical therapy**

| All syndromes combined (RTI, IAI, UTI, BSI; n=234; modelled on 230 as 4 were non-assessable) | | |
| --- | --- | --- |
| Outcome | Unadjusted OR (95% CI) | Adjusted OR for age, sex, CCI |
| ICU admission | 0.65 (0.27–1.57), p=0.339 | 0.64 (0.27–1.55), p=0.322 |
| Graft rejection within 30 days | 0.44 (0.04–4.98), p=0.508 | 0.41 (0.03–4.87), p=0.482 |
| Graft loss within 30 days | 0.66 (0.07–6.55), p=0.727 | 0.68 (0.07–7.05), p=0.745 |
| CDI with 30 days | -- | -- |
| Re-admission within 30 days | 1.12 (0.55–2.31), p=0.750 | 1.15 (0.56–2.37), p=0.708 |
| Mortality at 30 days | 0.37 (0.10–1.31), p=0.124 | 0.33 (0.09–1.23), p=0.099 |
| Composite outcome* | 0.74 (0.38–1.45), p=0.380 | 0.74 (0.38–1.50), p=0.388 |
|  | Unadjusted coefficient (95% CI) | Adjusted coefficient (95% CI) |
| Hospital LOS | 9.82 (-11.80–31.44), p=0.372 | 8.23 (-13.38–29.84), p=0.454 |

**RTI, empirical therapy**

| Respiratory tract infection (n=86) | | |
| --- | --- | --- |
| Outcome | Unadjusted OR (95% CI) | Adjusted OR for age, sex, CCI |
| ICU admission | 1.18 (0.13–10.65), p=0.882 | 1.19 (0.13–11.28), p=0.877 |
| Graft rejection within 30 days | -- | -- |
| Graft loss within 30 days | -- | -- |
| CDI within 30 days | -- | -- |
| Re-admission within 30 days | 0.48 (0.10–2.34), p=0.366 | 0.50 (0.10–2.53), p=0.402 |
| Mortality at 30 days | 0.21 (0.03–1.29), p=0.092 | 0.17 (0.02–1.37), p=0.096 |
| Composite outcome | 0.48 (0.10–2.31), p=0.364 | 0.41 (0.08–2.09), p=0.286 |
|  | Unadjusted coefficient (95% CI) | Adjusted coefficient (95%) |
| Hospital LOS | -9.95 (-43.33–23.43), p=0.555 | -11.03 (-45.7–23.60), p=0.528 |

**IAI, empirical therapy**

| Intra-abdominal infection (n=40 but model on n=39 as one was adjudicated as not assessable) | | |
| --- | --- | --- |
| Outcome | Unadjusted OR | Adjusted OR for age, sex, CCI |
| ICU admission | 0.40 (0.05–3.24), p=0.391 | 0.16 (0.01–2.28), p=0.178 |
| Graft rejection within 30 days | -- | -- |
| Graft loss within 30 days | -- | -- |
| CDI with 30 days | -- | -- |
| Re-admission within 30 days | 8.80 (0.99–78.1), p=0.051 | 9.60 (0.99–92.83), p=0.051 |
| Mortality at 30 days | -- | -- |
| Composite outcome | 1.86 (0.45–7.67), p=0.392 | 1.50 (0.33–6.86), p=0.602 |
|  | Unadjusted coefficient (95% CI) | Adjusted coefficient (95% CI) |
| Hospital LOS | 13.73 (-15.31–42.77), =0.344 | 13.56 (-17.83–44.95), p=0.386 |

**UTI, empirical therapy**

| Urinary tract infection (n=82) | | |
| --- | --- | --- |
| Outcome | Unadjusted OR | Adjusted OR for age, sex, CCI |
| ICU admission | 0.47 (0.08–2.65), p=0.392 | 0.24 (0.04–1.69), p=0.153 |
| Graft rejection within 30 days | -- | -- |
| Graft loss within 30 days | -- | -- |
| CDI with 30 days | -- | -- |
| Re-admission within 30 days | 0.61 (0.14–2.63), p=0.506 | 0.64 (0.14–2.90), p=0.566 |
| Mortality at 30 days | -- | -- |
| Composite outcome | 0.25 (0.05–1.34), p=0.107 | 0.22 (0.04–1.22), p=0.083 |
|  | Unadjusted coefficient (95% CI) | Adjusted coefficient (95% CI) |
| Hospital LOS | 18.10 (-44.75–80.96), p=0.568 | 9.54 (-54.76–73.84), p=0.769 |

**Bacteremia, empirical therapy**

| Bacteremia (total n = 38 but model n=35 because 3 were adjudicated as not assessable) | | |
| --- | --- | --- |
| Outcome | Unadjusted OR | Adjusted OR for age, sex, CCI |
| ICU admission | 1.00 (0.19–5.33), p=1.00 | 0.97 (0.17–5.58), p=0.974 |
| Graft rejection within 30 days | 0.74 (0.04–12.82), p=0.834 | 0.76 (0.04–15.79), p=0.858 |
| Graft loss within 30 days | -- | -- |
| CDI with 30 days | -- | -- |
| Re-admission within 30 days | 2.44 (0.61–9.80), p=0.207 | 3.28 (0.72–15.03), p=0.125 |
| Mortality at 30 days | 0.72 (0.09–5.81), p=0.760 | 0.77 (0.07–9.39), p=0.857 |
| Composite outcome | 2.67 (0.66–10.75), p=0.168 | 3.81 (0.80–18.19), p=0.094 |
|  | Unadjusted coefficient (95% CI) | Adjusted coefficient (95% CI) |
| Hospital LOS | 36.72 (-17.16–90.59), p=0.175 | 48.2 (-4.09–100.4), p=0.069 |

**Outcomes, tailored therapy:**

**All syndromes, tailored therapy**

| All syndromes combined (RTI, IAI, UTI, BSI, CDI) | | |
| --- | --- | --- |
| Outcome | Unadjusted OR (95% CI) | Adjusted OR for age, sex, CCI |
| ICU admission | 0.35 (0.10–1.18), p=0.090 | 0.51 (0.13–1.97), p=0.328 |
| Graft rejection within 30 days | -- | -- |
| Graft loss within 30 days | -- | -- |
| CDI with 30 days | -- | -- |
| Re-admission within 30 days | 1.19 (0.36–3.93), p=0.769 | 1.25 (0.38–4.18), p=0.708 |
| Mortality at 30 days | 0.144 (0.03–0.61), p=0.008 | 0.07 (0.01–0.38), p=0.002 |
| Composite outcome | 0.75 (0.25–2.20), p=0.601 | 0.70 (0.24–2.09), p=0.526 |
|  | Unadjusted coefficient (95% CI) | Adjusted coefficient (95% CI) |
| Hospital LOS | 2.8 (-30.7–36.4), p=0.868 | -0.4 (-34.1–33.3), p=0.981 |

**RTI, tailored therapy**

| Respiratory tract infection (n=86) | | |
| --- | --- | --- |
| Outcome | Unadjusted OR (95% CI) | Adjusted OR for age, sex, CCI |
| ICU admission | 0.57 (0.05–5.86), p=0.633 | 0.67 (0.06–8.09), p=0.754 |
| Graft rejection within 30 days | -- | -- |
| Graft loss within 30 days | -- | -- |
| CDI with 30 days | -- | -- |
| Re-admission within 30 days | 1.17 (0.12–11.83), p=0.894 | 1.24 (0.11–13.94), p=0.864 |
| Mortality at 30 days | 0.28 (0.03–3.06), p=0.297 | 0.14 (0.01–2.56), p=0.186 |
| Composite outcome | 0.67 (0.09–5.02), p=0.700 | 0.45 (0.05–3.74), p=0.458 |
|  | Unadjusted coefficient (95% CI) | Adjusted coefficient (95% CI) |
| Hospital LOS | 14.4 (-28.93–57.71), p=0.511 | 14.96 (-31.10–61.02), p=0.520 |

**IAI, tailored therapy**

| Intra-abdominal infection (n=40) = all patients were concordant with guideline | | |
| --- | --- | --- |
| Outcome | Unadjusted OR (95% CI) | Adjusted OR for age and sex (95% CI) |
| ICU admission | -- | -- |
| Graft rejection within 30 days | -- | -- |
| Graft loss within 30 days | -- | -- |
| CDI with 30 days | -- | -- |
| Re-admission within 30 days | -- | -- |
| Mortality at 30 days | -- | -- |
| Composite outcome | -- | -- |
|  | Unadjusted coefficient (95% CI) | Adjusted coefficient (95% CI) |
| Hospital LOS | -- | -- |

***All IAI treatment were guideline concordant.**

**UTI, tailored therapy**

| Urinary tract infection (n=82) | | |
| --- | --- | --- |
| Outcome | Unadjusted OR | Adjusted OR (for age and sex) |
| ICU admission | 1.03 (0.11–9.42), p=0.978 | 1.02 (0.10–10.36), p=0.990 |
| Graft rejection within 30 days | -- | -- |
| Graft loss within 30 days | -- | -- |
| CDI with 30 days | -- | -- |
| Re-admission within 30 days | 1.03 (0.11–9.42), p=0.978 | 1.02 (0.10–10.36), p=0.990 |
| Mortality at 30 days | -- | -- |
| Composite outcomes | 2.31 (0.42–12.65), p=0.335 | 2.12 (0.37–11.99), p=0.396 |
|  | Unadjusted coefficient (95% CI) | Adjusted coefficient (95% CI) |
| Hospital LOS | -5.82 (-72.7–61.05), p=0.863 | -6.88 (-74.94–61.18), p=0.841 |

**Bacteremia, tailored therapy**

| Bacteremia (n=38) | | |
| --- | --- | --- |
| Outcome | Unadjusted OR | Adjusted OR (for age and sex) |
| ICU admission | 0.41 (0.03–5.33), p=0.499 | 0.15 (0.01–3.07), p=0.216 |
| Graft rejection within 30 days | -- | -- |
| Graft loss within 30 days | -- | -- |
| CDI with 30 days | -- | -- |
| Re-admission within 30 days | 1.68 (0.14–20.33), p=0.682 | 1.72 (0.12–23.70), p=0.685 |
| Mortality at 30 days | 0.03 (0.02–0.49), p=0.014 | -- |
| Composite outcomes | -- | -- |
|  | Unadjusted coefficient (95% CI) | Adjusted coefficient (95% CI) |
| Hospital LOS | 26.95 (-66.32–120.22), p=0.561 | 46.54 (-48.54–141.62), p=0.327 |

| Nosocomial C. difficile infection (n=29) | | |
| --- | --- | --- |
| Outcome | Unadjusted OR | Adjusted OR (for age and sex) |
| ICU admission | -- | -- |
| Graft rejection within 30 days | -- | -- |
| Graft loss within 30 days | -- | -- |
| CDI with 30 days | -- | -- |
| Re-admission within 30 days | -- | -- |
| Mortality at 30 days | -- | -- |
| Composite outcomes | -- | -- |
|  | Unadjusted coefficient (95% CI) | Adjusted coefficient (95% CI) |
| Hospital LOS | -- | -- |

***All nosocomial CDI treatment were guideline concordant.**
